# Off-Stoichiometry Driven Carrier Density Variation at the Interface of LaAlO_3_/SrTiO_3_

**DOI:** 10.1038/s41598-017-02039-x

**Published:** 2017-05-11

**Authors:** Ming-Shiu Tsai, Chi-Sheng Li, Shih-Ting Guo, Ming-Yuan Song, Akhilesh Kr. Singh, Wei-Li Lee, M.-W. Chu

**Affiliations:** 1 Institute of Physics, Academia Sinica, Nankang, Taipei, 11529 Taiwan; 2 Center for Condensed Matter Sciences, National Taiwan University, Taipei, 10617 Taiwan

## Abstract

The interface between LaAlO_3_ (LAO) and SrTiO_3_ (STO) has attracted enormous interests due to its rich physical phenomena, such as metallic nature, magnetism and superconductivity. In this work, we report our experimental investigations on the influence of the LAO stoichiometry to the metallic interface. Taking advantage of the oxide molecular beam epitaxy (MBE) technique, a series of high quality LAO films with different nominal La/Al ratios and LAO thicknesses were grown on the TiO_2_-terminated STO substrates, where systematic variations of the LAO lattice constant and transport property were observed. In particular, the sheet density can be largely reduced by nearly an order of magnitude with merely about 20% increase in the nominal La/Al ratio. Our finding provides an effective method on tuning the electron density of the two-dimensional electron liquid (2DEL) at the LAO/STO interface.

## Introduction

The discovery of 2DEL at the interface of two insulating diamagnetic oxides has a great potential due to its unique and interesting behaviour, such as metallic interface^1, 2^, magnetism^3–5^, superconductivity^6–8^ and unexpectedly coexistence of later two^9, 10^. However, the fundamental mechanism involved behind the origin of these properties is still debatable. Other than the known polar catastrophe mechanism^11–13^, the inter-atomic diffusion at the interface^14–17^ and electron doping via oxygen vacancies^2, 18–21^ are both likely sources for the metallic interface. More recently, the strain-induced polarization near the oxide interface may also play an important role^22–25^, which opens further possibilities in the oxide base electronics from both fundamental and application point of view.

On the other hand, there are several reports about the composition effects on 2DEL and related electrical and magnetic properties^26–30^, where the LAO films with different La/Al ratios were grown using pulse laser deposition (PLD)^27, 29, 30^, magnetron sputtering^28^ and MBE techniques^26^. In an oxide MBE system, the variation of LAO composition can be achieved by growing films at slight different sample locations with respect to the La and Al effusion cells, and Warusawithana *et al*.^26^ have reported that the La/Al ratio ≤ 0.97 ± 0.3 is a necessity for obtaining a 2DEL at the LAO/STO interface and LAO films exhibit insulating nature if La/Al is above 1. Their results faded out the extrinsic causes for 2DEL formation, such as oxygen vacancies occur either during film growth or preparation of TiO_2_-terminated STO substrate and inter-atomic diffusion of La across the interface into STO substrate. In contrast, different La/Al ratios can also be achieved by adjusting the laser fluence in the PLD film-growth process. Sato *et al*.^27^ reported that the off-stoichiometry films, regardless of La-rich or La-deficient, have reduced carrier density, and the sheet resistance for La-deficient samples are generally lower than that for La-rich samples. They attributed the reduction in the carrier density to the electronic and atomic reconstruction in the off-stoichiometry films. A comparison of the above results indicated that the chemical composition of LAO, largely affected by the growth technique and parameters, is crucial for the resulting electronic properties at the LAO/STO interface. In this work, we used the oxide-MBE technique to grow high quality epitaxial LAO films on STO substrate with different thicknesses and also different compositions by controlling the shutter-open time for La and Al effusion cells. Through careful structural and chemical characterizations and low temperature magneto-transport measurements, we found the La content in LAO layer has dramatic influence on the 2DEL at the LAO/STO interface. Our finding provides an effective method for tuning the electronic property of 2DEL at the oxide interface.

## Experimental Methods

All the LAO films were grown on atomically flat TiO_2_-terminated (100) STO substrates that were prepared using aqua regia followed by annealing at 1000 °C for 10 hours in pure oxygen at atmospheric pressure. Subsequently, STO substrates were loaded in the oxide MBE chamber under a base pressure better than 1.5 × 10^−10^ torr and then pre-annealed at 900 °C for about 20 minutes. The stoichiometric LAO films were grown by setting the sample temperature at 800 °C and observing the *in*-*situ* real time reflection high energy electron diffraction (RHEED) oscillation and images. The atomic composition of LAO film was further controlled by changing the shutter-open times for La source and Al source, where the corresponding atomic fluxes were calibrated in advance via quartz crystal microbalance and atomic absorption spectroscopy. In order to minimize possible oxygen loss in STO substrates during the pre-annealing and film-growth processes, the ozone partial pressure was always maintained at around 1 × 10^−6^ torr as long as the sample temperature was above 200 °C. The as-grown films were then *ex*-*situ* post annealed at 400 °C for 1 hour in pure oxygen at atmospheric pressure.

The surface morphology is observed by atomic force microscopy (AFM). The structural investigations were carried out using a single crystal X-ray diffractometer (XRD) and scanning transmission electron microscope (STEM). The compositional analysis was further characterized using the Rutherford backscattering spectrometry (RBS). For the electrical contacts fabrication, dry argon ion milling was first used to etch the sample to a depth below the interface with a pattern defined by a stencil mask, and it was then followed by the deposition of Ti/Au thin films as contact electrodes. The low temperature resistivity and Hall measurements were then carried out using a variable temperature insert (VTI) in a superconducting magnet system.

**Figure 1:**
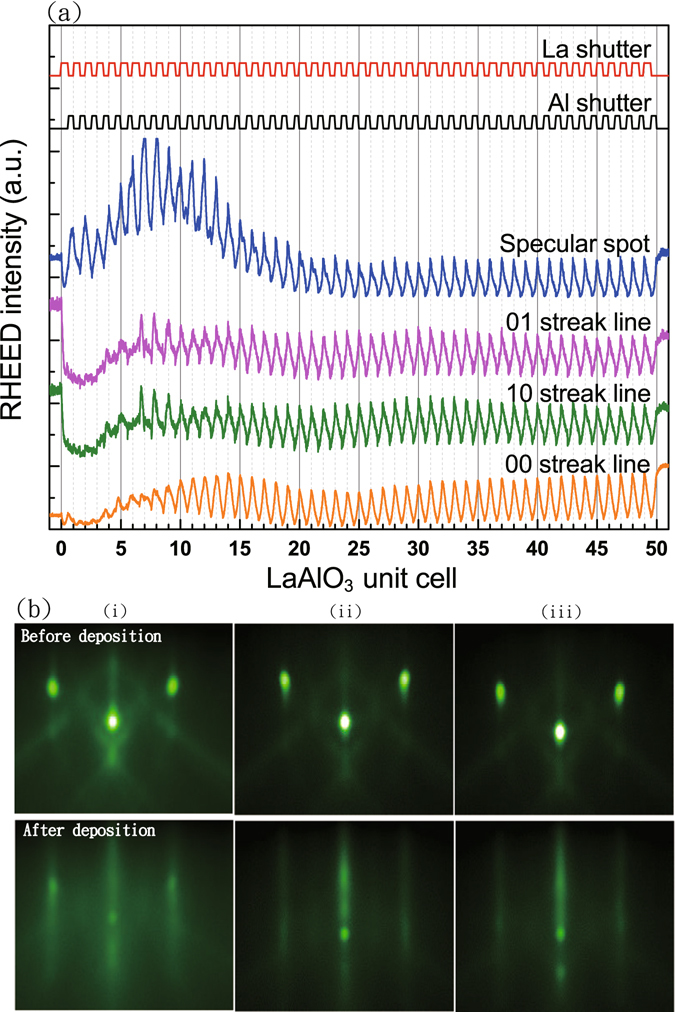
(**a**) RHEED oscillations of a 50-uc thin LAO film grown on the STO substrate. (**b**) The RHEED images along the STO[011] azimuth direction before (upper panel) and after (lower panel) the growth of LAO films with La/Al ratios = 1.1 (i), 1.0 (ii), and 0.9 (iii). The 3D spot-like feature evolves into 2D streak lines after the LAO film growth, indicating an atomic-scale uniformity of the grown ultra-thin LAO films without island formation.

Figure 1(a) displays *in*-*situ* real time RHEED oscillations of a 50 unit cells (uc) thick LAO film, which was used as a reference of stoichiometric LAO film with a La/Al ratio of 1.0. Therefore, 10% increase (decrease) of La shutter-open time results in a nominal La/Al ratio of 1.1 (0.9) in the as-grown LAO film. It should be noted that La and Al composition is also measured with RBS. However, it is a known issue that Al has a smaller backscattering cross-section, and its signal was buried in strong background from Sr and Ti^23, 31, 32^, which makes it difficult to determine the exact content of Al. Nevertheless, from the relative peak intensity and fittings (see supplementary information), we found the La/Al ratios inferred from RBS do qualitatively agree with the nominal La/Al ratios determined from the shutter-open time. The RHEED images before and after the LAO deposition for different La/Al ratio is shown in Fig. 1(b). The RHEED oscillation is not uniform up to 5-uc LAO possibly due to the strain effect originating from the lattice mismatch (see Fig. 1(a)) at the interface. However, for a thickness larger than 5-uc, intensity of the RHEED oscillation increases and becomes uniform for all the layers, revealing an atomic layer-by-layer growth of epitaxial LAO thin films. Moreover, the RHEED images (see Fig. 1(b)) along the STO[011] azimuth direction show a clear evolution from a spot-like feature of (100) STO substrate to long and thin streak lines for all the samples with different La/Al ratios, owing to the formation of broadened reciprocal lattice rods from ultra-thin LAO films.

**Figure 2:**
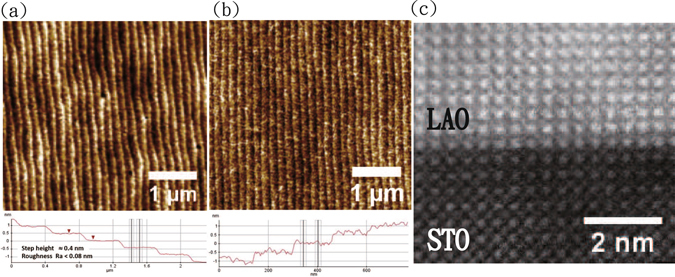
AFM images of a TiO_2_-terminated STO substrate before (**a**) and after (**b**) growth of 20-uc LAO film for La/Al ratio = 1.0 sample. Lower panels show the cross-sectional profiles of the atomic steps on the surface of samples. No significant change in the roughness and terraces is observed indicating the great smoothness and uniformity of the LAO films, which is also confirmed from the atomic structure revealed by the STEM image across the LAO/STO interface as shown in (**c**).

**Figure 3:**
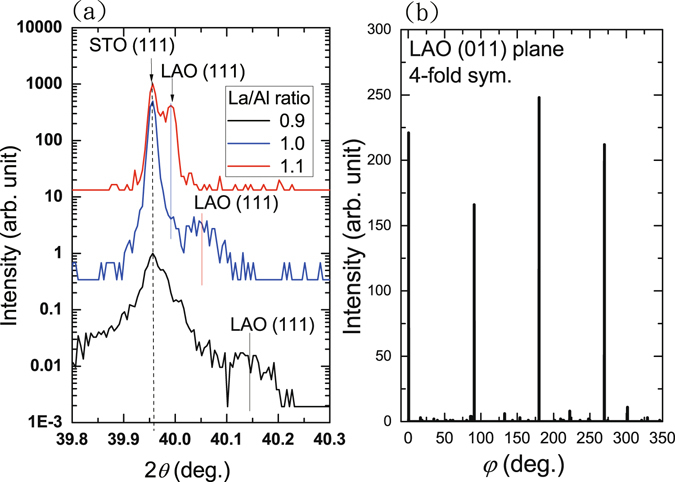
(**a**) *θ*-2*θ* XRD scans of the 100-uc LAO films with different La/Al ratios. (**b**) *φ*-scan of a 100-uc thin LAO film shows a fourfold symmetry.

Figure 2 displays the AFM images of TiO_2_-terminated STO substrate before and after the LAO film growth for La/Al ratio = 1.0 sample. The TiO_2_-terminated STO substrate displays atomic terraces with an average step height of 0.4 nm and an average surface roughness of around 80 pm. The typical width of the terraces varied from 100 nm to 300 nm. After growth of 20-uc LAO film (see Fig. 2(b)), no significant changes in the terraces width and surface roughness were found in the cross-sectional profiles as demonstrated in lower panels of Fig. 2(a,b) (see also supplementary information). It indicates the atomic uniformity and smoothness in the LAO films we grew, which was also supported by the STEM images for La/Al ratio = 1.0 sample shown in Fig. 2(c), exhibiting a nearly perfect atomic structure across the interface. Moreover, no significant change in the surface roughness and morphology was observed for films with different La/Al ratios (see supplementary information). Figure 3(a) illustrates the XRD pattern of LAO 100-uc thick films with different nominal La/Al ratios that were determined from the shutter-open times for La and Al sources during the film growth. In order to resolve the lattice variation, a single crystal X-ray diffractometer was used. The samples were tilted such that the (111) planes of both STO and LAO can be clearly identified (see supplementary information). We found that the LAO (111) peak position progressively shifted away from the STO (111) peak as the La/Al ratio decreases from 1.1 to 0.9, suggesting a significant strain relaxation in the LAO films with a smaller La/Al ratio^31^. The corresponding *φ* scans for all three different La/Al ratios samples, as shown in Fig. 3(b), exhibit a clear fourfold symmetry in good agreement with the expected cubic perovskite crystal structure.

## Results and Discussions

**Figure 4:**
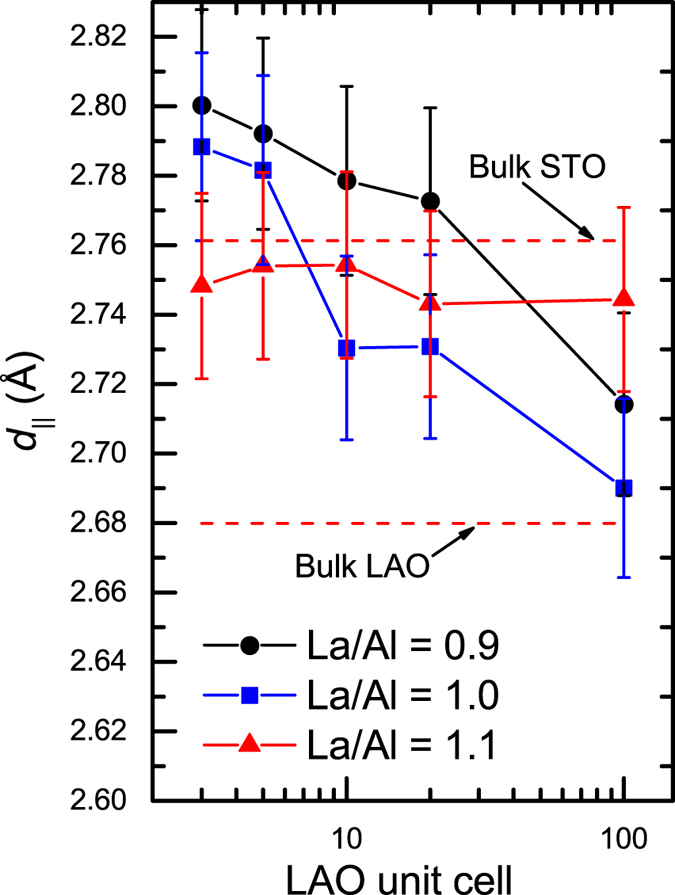
Variations of interatomic spacing *d*_‖_ of the LAO film alone the STO[011] azimuth direction with LAO film thickness for different La/Al ratios. The dashed lines are the *d*_‖_ values for bulk STO and bulk LAO.

By carefully analysing the RHEED images, the interatomic spacing *d*_‖_ between atomic rows parallel to the electron beam along the STO[011] azimuth direction can be extracted using the relation of *d*_‖_ · *b* = constant = *L* · *λ*_*e*_, where *b* is the streak lines spacing as shown in Fig. 1(b), *L* is the distance from the samples to RHEED screen, and *λ*_*e*_ is the electron wavelength. The resulting *d*_‖_ versus LAO film thickness for three different La/Al ratios is shown in Fig. 4, where the error bars come from the pixel resolution of the camera. For La/Al ratio = 0.9 and 1.0, *d*_‖_ is gradually marching down toward the bulk LAO *d*_‖_ value shown as a dashed line in Fig. 4. However, we remark that, for La/Al ratio = 1.1 samples, *d*_‖_ does not vary significantly up to a thickness of 100-uc, giving rise to a bigger lattice spacing at 100-uc compared to La/Al ratio = 0.9 and 1.0 samples with same thickness. This is in good agreement with the X-ray data shown in Fig. 3(a).

**Figure 5:**
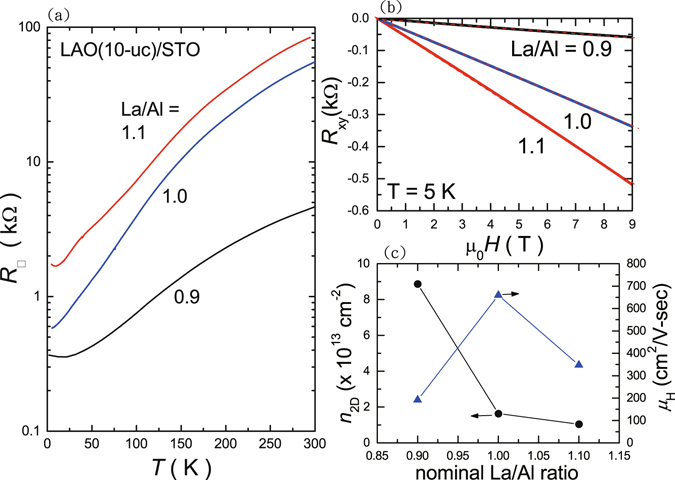
(**a**) Sheet resistance ${R}_{\square }$ versus temperature of 10-uc thin LAO films with different La/Al ratios. (**b**) The Hall resistance as a function of field at T = 5 K. (**c**) The corresponding sheet density *n*_2*D*_ and Hall mobility *μ*_*H*_ versus the nominal La/Al ratio.

Figure 5(a) illustrates the temperature dependence of sheet resistance (${R}_{\square }$) for 10-uc thick LAO films with different La/Al ratios. The ${R}_{\square }$-*T* curve for La/Al ratio = 1.0 sample exhibits metallic nature down to the lowest temperature of 2 K, while the sheet resistances for La/Al ratio = 1.1 and 0.9 samples start to increase with decreasing temperature below *T* $\simeq$ 10 K and 20 K, respectively. The ${R}_{\square }$ values at *T* = 5 K systematically decreases from 1,724 Ω for La/Al ratio = 1.1 to 367 Ω for La/Al ratio = 0.9. The corresponding residual resistivity ratios $({\rm{RRR}}\equiv {R}_{\square }(300\,{\rm{K}})/{R}_{\square }(5\,{\rm{K}}))$ for La/Al ratios of 1.1, 1.0 and 0.9 are of about 55, 95 and 13, respectively. The Hall resistance at *T* = 5 K is practically linear with field up to 9 Tesla as shown in Fig. 5(b). The normal Hall coefficient progressively increases with increasing value of La/Al ratio, suggesting a rapid decrease of the sheet density *n*_2*D*_ with increasing La/AL ratio. In Fig. 5(c), *n*_2*D*_ at *T* = 5 K equals 1.04 × 10^13^ cm^−2^ and 1.63 × 10^13^ cm^−2^ for La/Al ratio = 1.1 and 1.0, respectively, and it further increases by nearly an order of magnitude up to 8.87 × 10^13^ cm^−2^ for La/Al ratio = 0.9. We remark that the estimated Hall mobility *μ*_*H*_ reaches a maximum of about 660 cm^2^/V-s for La/Al ratio = 1.0, and it falls down for either La-rich or La-deficient samples as demonstrated in Fig. 5(c). The observation of the maximum values in both RRR and *μ*_*H*_ in our La/Al ratio = 1.0 sample further supports for a nearly perfect stoichiometry that we determined via comparing the *in*-*situ* RHEED oscillation profiles. In a recent work of sheet density tuning via ionic liquid gating^33^, we note that similar correlation between the *n*_2*D*_ and *μ*_*H*_ was observed.

The large variation in the ${R}_{\square }$ and *n*_2*D*_ with La/Al ratio in Fig. 5(c) is less likely due to oxygen vacancy effect, since the film-growth and post-annealing conditions were kept the same for all the samples we grew with different La/Al ratios. In addition, the trend of rapid decrease of *n*_2*D*_ in La-rich sample also excluded the possibility of La-doping effect on STO at the interface, where an opposite trend would have been expected. Several other possible mechanisms have been proposed previously to elucidate the influence of the La/Al ratio to the 2DEL at the LAO/STO interface, including the possible atomic and electronic reconstructions due to vacancies^26, 27^. On the other hand, we remark that the strain-related effect may also play an important role for the large *n*_2*D*_ variation with La/Al ratio, where observable changes in the lattice parameter in LAO films were clearly identified by the X-ray diffraction (Fig. 3(a)) and RHEED image analysis (Fig. 4). It has been suggested that the occurance of the metallic interface may be closely related to a slight volumn exapnsion in both LAO and STO near the interface^25^, where unusual ferroelectric-like polarizations appear, and the resulting domain wall at the interface becomes charge reservoirs. Such a feature seems to qualitatively agree with the slight larger *d*_‖_ value than bulk STO for thinner LAO samples with La/Al = 1.0 and 0.9 as shown in Fig. 4. On the contrary, for La/Al = 1.1 samples, *d*_‖_ is always below the bulk STO value for thickness down to 3 ML, and thus the ferroelectric-like polarizations may be strongly suppressed in La-rich samples, giving rise to a lower *n*_2*D*_ at the interface. Nevertheless, advanced characterizations of atomic-scale structure and chemical composition are beyond the scope of this work and require further investigations.

In summary, we have studied the effect of La/Al ratio on the 2-DEL and related properties of epitaxial LAO films grown by oxide MBE technique using pure ozone as oxidation agent. The composition of the LAO is readily controlled by adjusting La and Al shutter-open times, which was justified from the systematic variations observed in RBS and X-ray structural analysis. From the low *T* magneto-transport data, highest mobility and largest RRR were observed in the La/Al ratio = 1.0 sample as expected. Remarkably, the sheet density *n*_2*D*_ of the 2DEL at the LAO/STO interface increases by nearly an order of magnitude as the nominal La/Al ratio decreases by merely 20%. Our findings provide an effective method to tune the sheet density at the LAO/STO interface and may also shade some lights on the issue regarding the intrinsic mechanism for the formation of 2DEL at complex oxide interface.

## Electronic supplementary material

supplementary information
