# Supplementary material for: Off-Stoichiometry Driven Carrier Density Variation at the Interface of LaAlO3/SrTiO3
Source: Sci Rep. 2017 May 11;7:1770. doi: 10.1038/s41598-017-02039-x (PMC5431992; doi:10.1038/s41598-017-02039-x)
Supplement: Supplementary file 1 — supplementary information [file 41598_2017_2039_MOESM1_ESM.pdf]

Supplementary Information

Off-Stoichiometry Driven Carrier Density Variation at the Interface of LaAlO3/SrTiO3

Ming-Shiu Tsai, Chi-Sheng Li, Shih-Ting Guo, Ming-Yuan Song, M.-W. Chu, Akhilesh Kr. Singh and Wei-Li Lee

1. AFM images for LAO/STO samples with different La/Al ratios.

Supplementary figure S1 displays the AFM images of the $LaAlO_3$ films grown using molecular beam epitaxy technique. All the $LaAlO_3$ films exhibit clear terraces with the step height around 0.4 nm, which is very close to the TiO_2 terminated $SrTiO_3$ substrate. The AFM images revealed no significant change in the surface morphology and film roughness with respect to change in the La/Al ratio by around 20%, indicating a high quality of the grown films with different La/Al ratio.

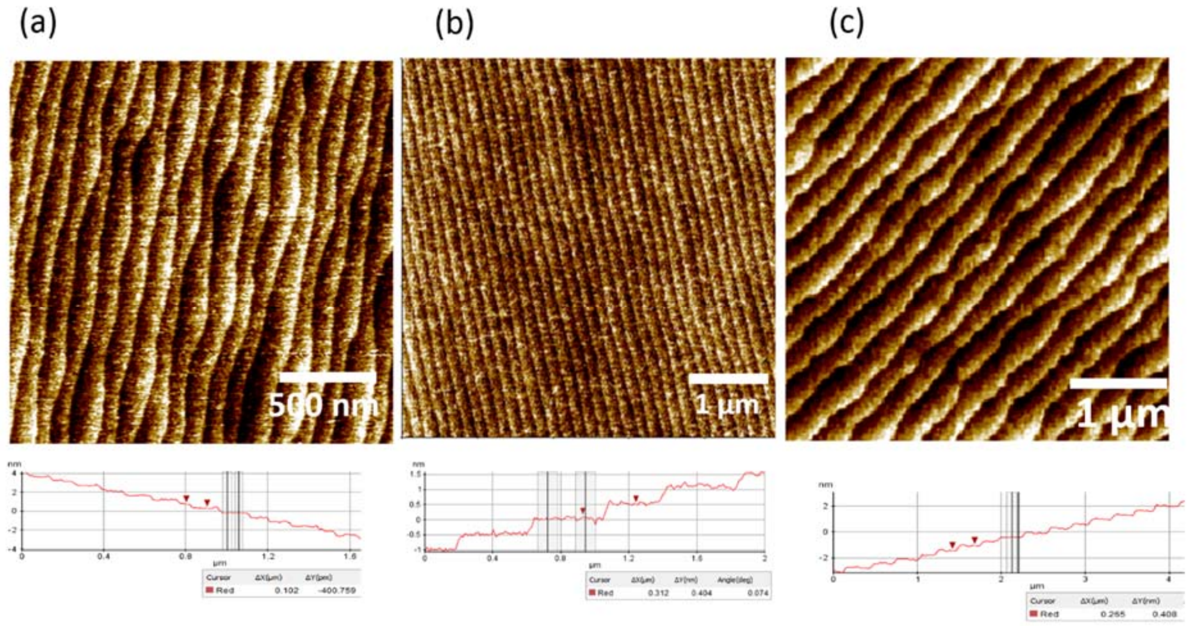

Supplementary Figure S1. The AFM image of the $LaAlO_3$ films grown on TiO_2 terminated (100) $SrTiO_3$ for (a) La/Al = 0.9, (b) for La/Al = 1.0 and (c) La/Al =1.1.

2. XRD measurements using single-crystal diffractometer.

Supplementary figure S2 illustrates the XRD patterns of the $LaAlO_3$ film grown on TiO_2 terminated (100) $SrTiO_3$ substrate. A single crystal X-ray diffractometer, having additional mechanism of sample stage rotation along any defined crystal plane, was used to measure the XRD patterns along different $LaAlO_3$ crystal planes. As demonstrated in the figure, we found that the XRD peak of $LaAlO_3$ along (111) plane turns out to be more prominent as compared to (001) and (011) planes. Moreover, the $LaAlO_3$ peak is shifted towards the $SrTiO_3$, indicating an increase in the lattice parameters of $LaAlO_3$. This also support for the existence of a sizable strain at the $LaAlO_3$/$SrTiO_3$interface.

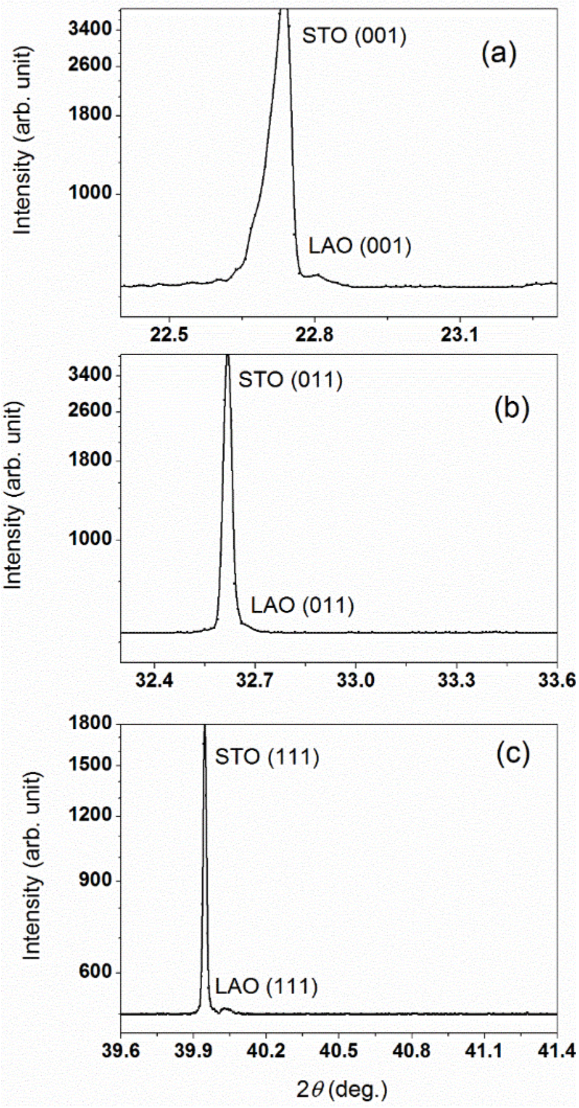

Supplementary Figure S2. XRD patterns of the $LaAlO_3$ films for La/Al = 1: (a) along (001), (b) along (011) and (c) along (111) planes of $LaAlO_3$ and $SrTiO_3$.

3. Rutherford backscattering spectroscopy (RBS) analysis.

Supplementary Figure S3 shows the RBS analysis of the $LaAlO_3$ films for different La/Al ratios. We used Li ions from an NEC 9SDH-2 3MeV Tandem Accelerator for the RBS measurements. The energy calibration was done on a gold film before taking the RBS spectrum on LAO/STO samples. As shown in the figure, the small hump near the 700 keV correspond to the backscattering signal from Al, which is embeded in large background signals from Sr and Ti. Therefore, the La/Al ratios obtained from the RBS analysis has larger uncertainty, and typically a different substrate (such as MgO) is needed for more precise atomic composition determination. Nevertheless, by performing peak function fitting to the RBS data, the resulting La/Al ratios are listed in the Suppementary Table1. We found that the La/Al ratios extracted from the from RBS data follow qualitatively the trend of nominal La/Al ratios that was controlled by the presise shutter opening time of La and Al during the growth. However, we do note

that the absolute values of La/Al ratio from RBS analysis are indeed showing larger variations compared to the nominal values, where high precision La/Al ratio determination from RBS requires more rigorous measurments using different substrate materials.

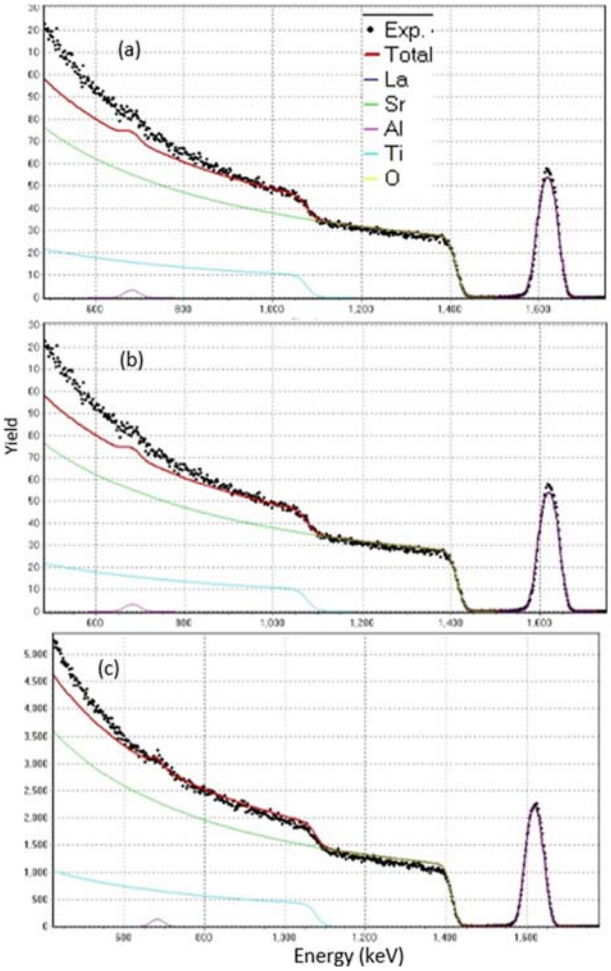

Supplementary Figure S3. The experimental RBS data along with fitting for the $LaAlO_3$ films grown on TiO_2 terminated $SrTiO_3$ substrate for different nominal La/Al ratios. (a) for La/Al = 0.9, (b) for La/Al = 1 and (c) for La/Al = 1.1.

Supplementary table 1. The comparison of nominal La/Al ratio with RBS La/Al ratio. The RBS results show systematic variation with the nominal La/Al ratios. The difference in the absolute value of La/Al ratio is due to the smaller backscattering cross-section of Al as compared to Sr and Ti.

Nominal La/Al ratio	0.9	**1**	**1.1**
RBS La/Al ratio	0.82	1	1.3
